# Long-lasting chronic high load carriage of Epstein-Barr virus is more common in young pediatric renal transplant recipients

**DOI:** 10.1007/s00467-019-04401-9

**Published:** 2019-12-04

**Authors:** Susanne Westphal Ladfors, Jenny K. Lindahl, Sverker Hansson, Per Brandström, Rune Andersson, Marianne Jertborn, Magnus Lindh, Susanne Woxenius, Vanda Friman

**Affiliations:** 1 Department of Pediatrics, Queen Silvia Children’s Hospital, Sahlgrenska University Hospital, Region Västra Götaland, Gothenburg, Sweden; 2 Department of Pediatrics, Institute of Clinical Sciences, Sahlgrenska Academy, University of Gothenburg, Gothenburg, Sweden; 3 Department of Infectious Diseases, Sahlgrenska University Hospital, Region Västra Götaland, SE-416 85 Gothenburg, Sweden; 4 Department of Infectious Diseases, Institute of Biomedicine, Sahlgrenska Academy, University of Gothenburg, Gothenburg, Sweden; 5 Department of Clinical Microbiology, Sahlgrenska University Hospital, Region Västra Götaland, Gothenburg, Sweden

**Keywords:** Epstein-Barr virus. EBV DNA, Chronic high load carrier, Infection. Pediatric, Renal transplantation

## Abstract

**Background:**

Epstein-Barr virus (EBV) infections can induce post-transplant lymphoproliferative disorder (PTLD). A chronic high load (CHL), as indicated by long-term high EBV DNA levels after transplantation, has been associated with an enhanced risk of PTLD. We aimed to evaluate incidence, time of occurrence, risk factors, and outcome of EBV CHL carrier state after pediatric renal transplantation.

**Methods:**

A retrospective study of 58 children aged 1–17 years (median 10), who underwent renal transplantation between January 2004 and June 2017 at a single medical center. EBV IgG antibodies in serum were analyzed before and yearly after transplantation. EBV DNA in whole blood were analyzed weekly for the first 3 months post-transplant, monthly up to 1 year and then at least once yearly. CHL was defined as EBV DNA ≥ 4.2 log_10_ Geq/ml in > 50% of the samples during ≥ 6 months.

**Results:**

At transplantation, 31 (53%) patients lacked EBV IgG and 25 (81%) of them developed primary EBV infection post-transplant. Of the 27 seropositive patients, 20 (74%) experienced reactivation of EBV. Altogether, 14 (24%) children developed CHL, starting at a median of 69 days post-transplant and lasting for a median time of 2.3 years (range 0.5–6.5), despite reduction of immunosuppression. Patients with CHL were younger and 11/14 were EBV seronegative at transplantation. No child developed PTLD during median clinical follow-up of 7.8 years (range 0.7–13).

**Conclusions:**

CHL was frequent, long lasting, and occurred mainly in young transplant recipients. The absence of PTLD suggests that monitoring of EBV DNA to guide immunosuppression was effective.

## Introduction

Epstein-Barr virus (EBV) infection may constitute a serious risk for EBV-associated complications in transplant recipients, whose cellular and humoral response is compromised by immunosuppressive therapy [1, 2]. Post-transplant lymphoproliferative disorder (PTLD) develops due to uncontrolled proliferation of lymphocytes after solid organ (SOT) or hematopoietic stem cell transplantation [3, 4]. The vast majority of PTLD cases are EBV-related [5–8]. Previous studies have indicated that the incidence of PTLD is higher in children than in adults [9]. The risk for PTLD in the pediatric SOT population is highest after intestinal transplantation (26.8%), followed by heart-lung (19.5%), heart (7.7-12.9%), liver (4%), and kidney transplantation (1–7%) [10–18]. EBV-associated PTLD in pediatric renal transplant recipients has been associated with graft loss [19] and has a mortality rate of 32–48% [15, 20, 21]*.*

According to many studies, important risk factors for PTLD in pediatric graft recipients are the lack of EBV IgG antibodies at the time of transplantation, the overall burden of immunosuppressive therapy, the presence of a concomitant primary cytomegalovirus (CMV) infection and high EBV DNA levels in blood [7, 14, 16, 22–24]. However, in other studies, transplant recipients have been observed to display high EBV loads without developing PTLD [25, 26]. There is also conflicting data on whether a long-lasting period of high EBV load is a predictor for the later development of EBV-related PTLD. The incidence of chronic high EBV load carrier state, as defined by Green et al. [17], was 8% in a study of pediatric renal transplant recipients [27]. Despite the association between EBV infection and the risk of developing PTLD after pediatric renal transplantation, there is still no consensus on viral load monitoring or the benefits of EBV-specific antiviral treatment in this population.

The aim of our study was to evaluate the incidence, time of occurrence, risk factors, and outcome of EBV chronic high load (CHL) carrier state after pediatric renal transplantation.

## Methods

### Patients and data collection

We performed a retrospective, single-center study of children undergoing renal transplantation at the Queen Silvia Children’s Hospital, Sahlgrenska University Hospital, in Gothenburg, Sweden. Our renal transplantation program began in 1986 and since then more than 100 children have been transplanted. From 2004 and on, patients have been monitored post-transplant with quantitative PCR for EBV and CMV. All 58 children below 18 years of age who had their first renal transplant between January 2004 and June 2017 were included and followed regularly until February 2018. The patients were censored when reaching 18 years of age (*n* = 31), at the time of re-transplantation (*n* = 1), or at the time of death (*n* = 1).

All patients were tested for human leucocyte antigens (HLA-A, B, C, DR, and DQ). Transplant recipients were cross-matched against donors using complement-dependent cytotoxicity (CDC) assay and flow cytometric lymphocyte crossmatch. A positive CDC was a contraindication for transplantation.

Serological analyses of donors and recipients regarding EBV and CMV antibodies (EBV in donors since 2006**)** were performed, along with post-transplant serial measurements of EBV and CMV DNA levels. Patients were seen three times weekly during the first month, twice a week for the following 2 months, once a week up to 6 months, and once every other week until 1 year post-transplant. Thereafter, clinical visits were gradually tapered to every sixth to eighth week. The patients had follow-up appointments at our hospital at least once a year. Data were collected at these visits as well as from medical charts kept at local hospitals. Routine clinical status and laboratory tests, including serum creatinine and tacrolimus trough concentration in blood, were assessed at each clinical visit. Glomerular filtration rate (GFR) measured by chromium-51-ethylene diamine tetraacetic acid clearance was performed at 3 months, 1 year, and yearly post-transplant thereafter.

Using a clinical chart review, we systematically extracted data that included diagnosis, age at transplantation, gender, donor source, HLA mismatches, immunosuppressive regimen, antiviral medication, EBV and CMV serology, and DNA levels, as well as clinical symptoms of infections, GFR, and survival data.

### Immunosuppressive protocol

The initial immunosuppressive treatment is summarized in Table 1. The standard protocol included corticosteroids, calcineurin inhibitors (CNI; tacrolimus/cyclosporine A), and mycophenolate mofetil (MMF). All patients received induction therapy with methylprednisolone, which since 2010 was combined with two doses of interleukin-2-receptor antagonist on day 0 and day 4. Intravenous methylprednisolone was given peri-operatively in a dose of 600 mg/m^2^. Prednisolone was started with 60 mg/m^2^ at day 0 and tapered to 5 mg/m^2^ daily within the first 3 months, to 10 mg/m^2^ every other day within the following 3 months and to 5 mg/m^2^ every other day from 6 months post-transplant onwards. The dose was not regularly modified or stopped upon EBV-infection or reactivation. Tacrolimus was initially given in a dose of 0.2 mg/kg daily and then adjusted to maintain trough levels of 5 to 8 ng/ml in whole blood for the first 3 months, and 4 to 7 ng/ml thereafter. Prior to 2010, the target levels for tacrolimus were higher in the first months post-transplant (10 to 12 ng/ml).

**Table 1:** Patient characteristics

Characteristics	All patients *n* = 58 (100%)	EBV carrier state		*p* value
		CHL *n* = 14 (24%)	Non-CHL *n* = 44 (76%)	
Age at renal transplantation; median, (range) (year)	10 (1–17)	2 (1–15)	12 (2–17)	< 0.0001
Boys/girls	29/29	10/4	19/25	0.12
Diagnosis:				
CAKUT	25	10 (71%)	15 (34%)	0.021
Hereditary disorders	18	3 (22%)	15 (34%)	
Acquired diseases	13	1 (7%)	12 (27%)	
Unknown	2	–	2 (5%)	
No dialysis prior to tx	23	5 (36%)	18 (41%)	0.98
HLA mismatch 0–2	26	5 (36%)	21 (48%)	
3–4	23	8 (57%)	15 (34%)	
5–6	9	1 (7%)	8 (18%)	0.97
Living donor	44	13 (93%)	31 (70%)	0.17
Cold ischemic time; median, (range) (h)	2^a^ (1–18)	1.8^a^ (1.3–9)	2.1^a^ (1–18)	0.10
Initial immunosuppressive regimen:				
Interleukin-2 receptor antagonist	38	6	32	
Cyclosporine A	2	0	2	
Tacrolimus	56	14	42	
Mycophenolate mofetil	58	14	44	
Corticosteroids	58	14	44	
GFR; median (range) (ml/min/1.73 m^2^)				
3 months after tx	69 (25–114)	82 (51–114)	67 (25–103)	0.0016
1 year after tx	69^b^ (39–109)	76 (53–109)	67^b^ (39–96)	0.19
Post-tx follow-up time; median, (range) (year)	3.7 (0.4–13)	7.8 (0.7–13)	2.9 (0.4–11)	
Rejection	16	3	13	0.83
Second renal transplantation	1	1	0	
Ad mortem	1	0	1	

Values are expressed as number (%), unless specified. For categorical variables, *n* (%) is presented. For continuous variables median (min; max)/ is presented. For comparison between groups, Fisher’s exact test (lowest one-sided *p* value multiplied by 2) was used for dichotomous variables and the Mantel-Haenszel chi-square test was used for ordered categorical variables and chi-square test was used for non-ordered categorical variables and the Mann-Whitney *U* test was used for continuous variables.

*CHL* chronic high load, *non-CHL* non-chronic high load consisting of low viral load (LVL) and undetectable viral load (UVL), *CAKUT* congenital anomalies of the kidney and urinary tract, *GFR* glomerular filtration rate, *tx* transplantation

^a^Cold ischemic time for four patients were lacking (*n* = 54), two patients in the CHL group (*n* = 12) and two in the non-CHL group (*n* = 42)

^b^GFR-data for one patient at 1 year post-transplant missing because deceased (*n* = 57 in all patients and *n* = 43 in non-CHL)

Cyclosporine A trough levels in whole blood were maintained at 150 to 200 ng/ml. MMF was given in a dose of 600 mg/m^2^ daily. The dose was adjusted to meet mycophenolic acid area-under-the curve (MPA-AUC), with target levels of 40 to 60 mg per liter and hour [28].

Immunosuppression was assessed at each clinical visit and individually adjusted. When EBV or CMV DNA was detected, the DNA levels were surveilled more frequently and reduction of immunosuppression was considered when EBV or CMV DNA levels of ≥ 3 log_10_ Geq/ml were reached. Thereafter, a stepwise reduction of MMF and tacrolimus was carried out if the EBV DNA levels increased ≥ 0.5 log_10_ Geq/ml or levels above 4 log_10_ Geq/ml were observed. When rejection was suspected, a renal biopsy was performed, and bolus doses of methylprednisolone were given when rejection was confirmed. Since 2013 onwards, the development of donor-specific antibodies (DSAs) was monitored.

### Virological analyses

All virological analyses were performed by accredited diagnostic assays at the Department of Clinical Microbiology, Sahlgrenska University Hospital. EBV IgG, IgM, and CMV IgM antibodies were analyzed using immunofluorescence, whereas CMV IgG antibodies were analyzed by an enzyme-linked immunosorbent assay (ELISA). Both EBV IgG and IgM detect viral capsular antigens. Patients with an EBV or CMV IgM antibody titer of ≥ 80 indicated a primary infection. An EBV IgG antibody titer of ≥ 32 or a CMV IgG antibody titer of ≥ 200 was considered seropositive, indicating previous EBV or CMV antigen exposure. The patients and the donors were analyzed for EBV and CMV serology status pre-transplant, and determination of EBV and CMV antibodies in serum was performed 3 months post-transplant and then annually.

Serial measurements of EBV and CMV DNA load in blood samples were performed at least every week during the first 3 months, once monthly up to 1 year after transplantation and thereafter according to EBV and/or CMV PCR-status. When the levels of EBV or CMV DNA increased, when EBV or CMV infection was suspected, or when the patient was treated for rejection, samples were taken more often. EBV DNA-positive patients were subsequently evaluated at regular intervals. Patients with rapidly rising levels or high levels for a long time were carefully examined for signs of PTLD. Clinical evaluations, laboratory tests, ultrasounds, or CT-scans of suspected organs were performed and additional investigations such as biopsy of lymph nodes and specific organs were considered. The children were thereafter monitored weekly regarding clinical status, tacrolimus trough levels, serum levels of creatinine, and EBV DNA levels.

Serum and whole blood samples were analyzed for EBV and CMV DNA with a real-time quantitative PCR using primers and probes by Niesters et al. in 2002 [29]*.* The viral loads were calculated from the slope and intercept of the standard curve, and results were expressed as log_10_ genome equivalents (Geq) per ml. The lower detection limit for the assays are ≈ 2.3 log_10_ (≈ 200) Geq of EBV or CMV DNA per ml. The same assay has been used during the whole study period.

The criteria for EBV and CMV DNAemia, infections, and disease are presented in Table 2. EBV and CMV infection/disease was defined as described by Bingler et al. [30] and Ljungman et al. [31].

**Table 2:** Definitions of EBV and CMV infections

Categories	Definition/criteria
EBV or CMV DNAemia/infection	Detection of EBV and/or CMV DNA by PCR in serum or whole blood at least twice within a month
Primary EBV or CMV infection	Detection of viral DNA in serum, whole blood, any body fluid, or tissue specimen by PCR in a previously seronegative person
Reactivated EBV or CMV infection	Detection of EBV and/or CMV DNA in serum, whole blood, any body fluid or tissue specimen at least twice within a month in a previously seropositive individual
Asymptomatic infection	Presence of EBV and/or CMV DNA in serum, whole blood, any body fluid, or tissue specimen in the absence of symptoms or when symptoms were more likely due to other causes
Symptomatic EBV or CMV infection/disease	Presence of EBV and/or CMV DNA in serum, whole blood, any body fluid, or tissue specimen in combination with symptoms such as prolonged fever, malaise, night sweats, lymphadenopathy, pharyngitis, tonsillitis, and/or hepatitis, without histological evidence of PTLD or CMV tissue invasive disease
CMV tissue invasive disease	Detection of CMV DNAemia and evidence of organ involvement (hepatitis, gastrointestinal disease etc.), based on symptoms and/or pathology
EBV and CMV co-infection	Detection of CMV DNAemia in patients belonging to the EBV-groups LVL or CHL. CMV DNA should be detected within 1 month before, after, or at the same time as EBV DNA was detected.

### EBV DNA carrier states

The children were divided into two groups according to their EBV DNA levels: chronic high EB viral load (CHL) and non-chronic high load (non-CHL). CHL was defined as the presence of EBV DNA ≥ 4.2 log_10_ Geq/ml in whole blood, in > 50 % of the samples for ≥ 6 months as previously defined by Green et al. [17]. Non-CHL consisted of patients with undetectable EB viral load (UVL) and patients with low EB viral load (LVL). UVL was defined as having no more than one sample of detectable EBV DNA levels following transplantation, and low EB viral load (LVL) included children not meeting criteria for UVL nor CHL.

### Antiviral prophylaxis and treatment

All given blood products were leukocyte reduced. Antiviral prophylaxis with ganciclovir (before 2005) or valganciclovir (from 2005 onwards) was given to patients at high risk of primary CMV infection or at risk of CMV reactivation. Seronegative recipients received prophylaxis for 6 months in case of seropositive donor (D+/R−). Seropositive recipients (R+) received antiviral prophylaxis for 3 months. The prophylaxis was initiated about 7 days post transplantation. No prophylaxis was given to seronegative recipients with seronegative donors (D−/R−) except for four children who were considered to have an increased risk of contracting primary CMV-infection, such as having siblings in pre-school.

Antiviral treatment with ganciclovir or valganciclovir was given to patients with CMV DNA levels of ≥ 3 log_10_ Geq/ml, quickly rising CMV DNA levels, primary CMV infection, or when symptomatic CMV infection was suspected.

### Statistical methods

Age and calculated values for time after transplantation are expressed as median (range), unless specified. Categorical variables are described by number and percentage.

Mann-Whitney *U* test was used for comparisons between CHL and non-CHL with respect to continuous variables in Fig. 2. Mantel-Haenszel Chi-square test was used to test between the two groups (CHL and non-CHL) with respect to ordered categorical variables (for example age categories) and chi-square for non-ordered categorical variables (for example different diagnoses analyzed with one variable).

The incidence of CHL was described by crude event rates per 100 person-years, computed as number of events divided by number of follow-up time per 100 years. The 95% CIs were calculated using exact Poisson confidence limits. The impact of age at transplantation, sex, diagnosis, HLA mismatch, living donor, no dialysis before transplantation, and cold ischemic time on time to CHL were evaluated by univariable Cox proportional hazards models. Time-updated Cox regression was applied when studying the impact of primary infection/re-activation among all patients and primary infection among seronegative patients at transplantation. Hazard ratios (HRs) with 95% confidence intervals (CIs) are presented as effect sizes. The assumption of proportional hazards in the models was checked by investigating log (−log (survival)) vs. log (time) curves and by introducing an interaction term with log (time) in the model and was found satisfactory. The only independent significant predictor was age at transplantation; hence, no multivariable model was constructed.

All tests were two-tailed and conducted at a significance level of 0.05. The analyses were performed using SAS software version 9.4 (SAS Institute Inc., Cary, NC, USA).

## Results

### Patient characteristics

The characteristics of the 58 renal transplant patients are presented in Table 1. The age at transplantation ranged from 1 to 17 years (median 10). Half of the children were boys and 40% were transplanted without prior dialysis. Congenital anomalies of the kidney and urinary tract (CAKUT) and hereditary disorders were the most common underlying diseases. Living related donors were used in 44 patients (76%). The median donor age was 39 years (0.3–57). The median follow-up time post-transplant was 3.7 years (0.4–13) in the whole cohort. The cold ischemic time was 2 h (1–18). Fifteen patients (26%) developed acute cellular graft rejection, and one patient developed an early acute antibody-mediated rejection. There was no difference between the patients in the CHL group compared to the non-CHL group in immunosuppressive treatment or rejections. The initial immunosuppressive protocol and follow-up was the same in all children but was individualized during follow-up depending on EBV status. Median GFR 3 months post-transplant was 69 (25–114) and after 1 year 69 ml/min/1.73 m^2^ (39–109). Patients in the non-CHL group had a shorter follow-up time than patients in the CHL group. Thirty-one patients were censored when reaching 18 years of age, one patient at the time of re-transplantation 7.3 years post-transplant and the others at the end of follow-up. One patient died 9 months after transplantation due to bacterial pneumonia and multi-organ failure while having high EBV DNA levels (maximum level at 4.07 log_10_ Geq/ml, but not qualifying for CHL) and concomitant CMV DNAemia, which was treated with ganciclovir.

### EBV status

**Fig. 1:**
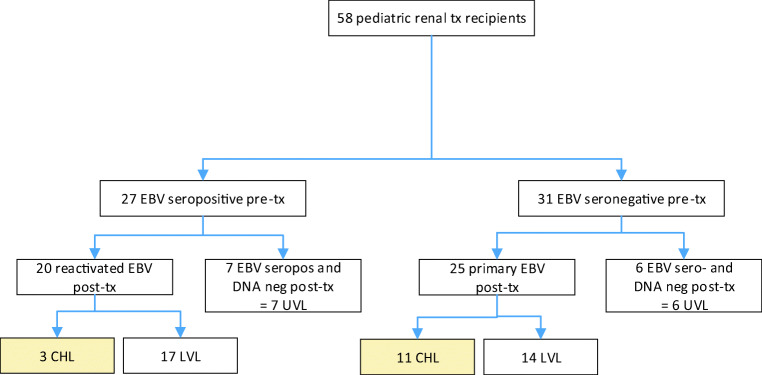
EBV outcome. Enrollment and follow up showing the pre-transplant EBV serostatus of renal transplant recipients and the development of EBV DNA positivity, i.e., chronic high load (CHL) and low viral load (LVL) and EBV DNA negativity, i.e., undetected viral load (UVL) during follow up. Tx transplantation

**Fig. 2:**
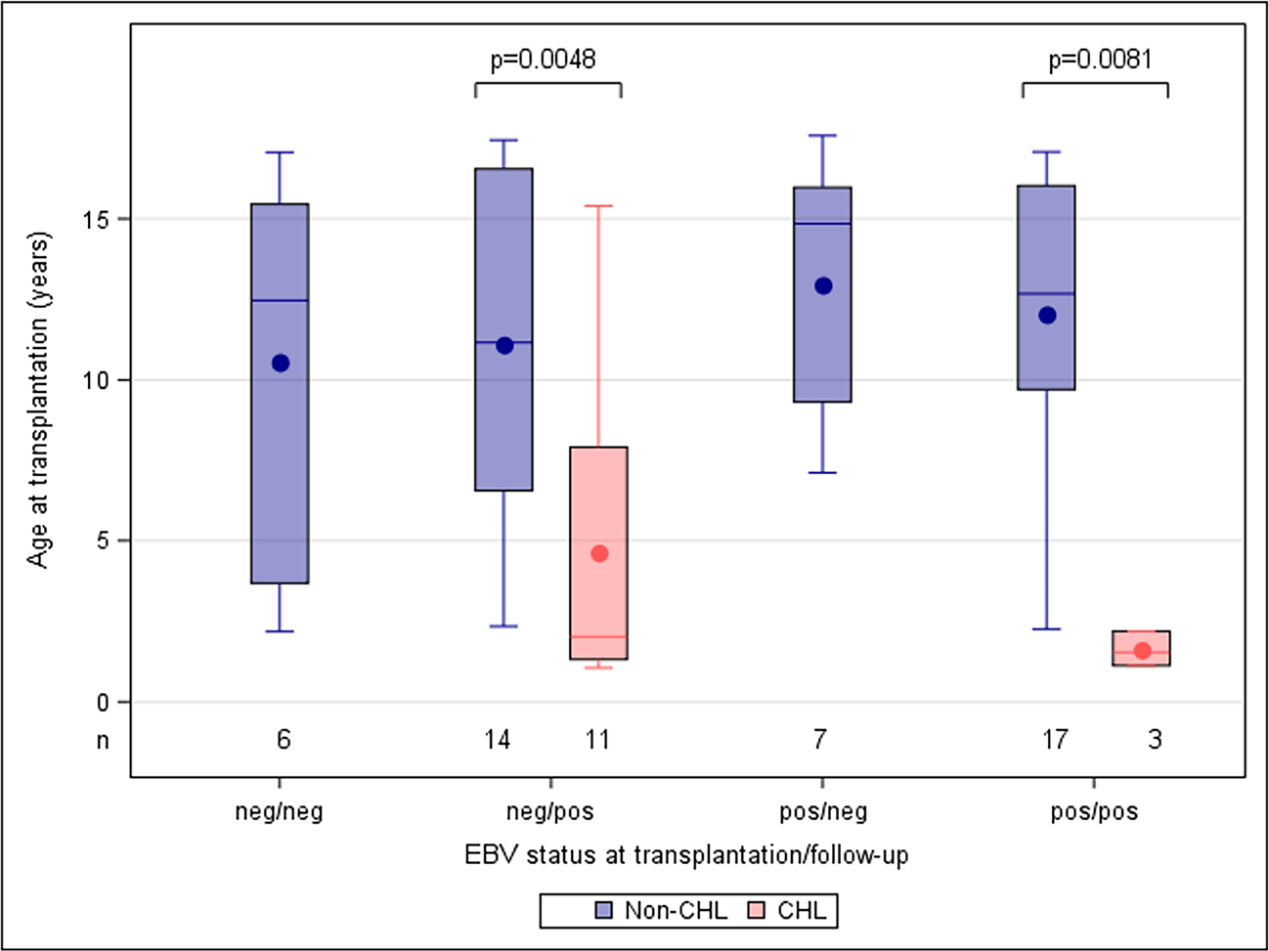
Distribution of age at transplantation for kidney recipients with different EBV status at transplantation/follow up, divided by those who developed chronic high EBV load (CHL) and non-CHL. Younger age and more EBV naive children at transplantation (tx) are seen in the CHL group in red compared to the non-CHL group in blue. Fourteen non-CHL and 11 CHL patients had a primary EBV infection. Seventeen non-CHL and three CHL patients had a reactivated EBV infection. Seven non-CHL patients that were EBV seropositive at transplantation stayed negative in EBV DNA measured by PCR method post-transplant

Thirty-one (53%) of the recipients were EBV seronegative before transplantation (Fig. 1; Table 3). Twenty-eight (90%) of them received an organ from an EBV-seropositive donor (D+/R−). Serostatus for the remaining three donors was unknown. Post-transplant, 25 (81%) of the seronegative recipients developed a primary EBV infection after a median of 43 days (14–363), while six patients remained EBV sero- and DNA-negative during follow up (Figs. 1 and 2).

**Table 3:** EBV and CMV characteristics of donors and recipients of renal transplants

Characteristics	All patients *n* = 58 (100%)	EBV carrier state	
		CHL *n* = 14 (24%)	Non-CHL *n* = 44 (76%)
EBV serostatus			
D^+^/R^−^	28	10 (71%)	18 (41%)
D^+^/R^+^	18	3 (21%)	15 (34%)
D^?^/R^+^	9	0	9 (20%)
D^?^/R^−^	3	1 (7%)	2 (5%)
CMV serostatus			
D^+^/R^-^	18	5 (36%)	13 (30%)
D^+^/R^+^	17	3 (21%)	14 (32%)
D^−^/R^+^	6	0	6 (14%)
D^−^/R^−^	17	6 (43%)	11 (25%)
EBV and CMV seropositive donor	27	7 (50%)	20 (45%)
EBV and CMV seropositive recipient	13	0	13 (30%)
EBV: DNAemia	45	14 (100%)	31 (70%)
Primary infection	25	11	14
Reactivated infection	20	3	17
Symptomatic infection	13	8	5
CMV: DNAemia	25	8 (57%)	17 (39%)
Primary infection	13	5	8
Reactivated infection	12	3	9
Symptomatic infection	10	6	4
Co-infection EBV and CMV	19	8 (18%)	11 (25%)
CMV prophylaxis			
None	13	2	11
3 months (CMV D−R+ or D+R+)	24^a^	5^b^	19^a^
6 months (CMV D+R−)	21^a^	7^a,b^	14^c^
Antiviral treatment of CMV	3	–	3

*CHL* chronic high load, *non-CHL* non-chronic high load consisting of low viral load and undetectable viral load, *D* donor serostatus, *R* recipient serostatus

^a^Ganciclovir as CMV prophylaxis post-transplant was given to two patients in the CHL group for 6 months (CHL 13 and 14 in Table 4) and to one patient in the LVL group for 3 months. All the other patients received valganciclovir

^b^Valganciclovir as CMV prophylaxis was given to four patients in the CHL group even though D−/R−, two for 3 months and two for 6 months because of increased risk of contracting primary CMV infection such as having siblings in pre-school

^c^Valganciclovir as CMV prophylaxis was given for 6 months to three patients in the non-CHL group even though D+/R+ because of increased risk of contracting primary CMV infection such as receiving anti-rejection treatment

Twenty-seven patients were EBV seropositive at transplantation, and in 20 (74%) of them, EBV reactivated after 12 days (0–64). EBV DNA in whole blood became negative in 13 of these patients after 3.2 years (0.1–8), while seven patients remained EBV DNA positive at last follow up. The seven patients who did not reactivate EBV post-transplant had a median age at transplantation of 14 years (7–17), for details see Fig. 2.

The initial immunosuppressive protocol was the same in all 58 patients. Among the 44 patients in the non-CHL-group, 13 experienced sporadic high EBV DNA loads: eight developed primary EBV infection and five a reactivated infection. These 13 were assumed to be at risk for developing CHL and PTLD, and therefore, the immunosuppression was reduced in the same way as for CHL carriers.

### EBV chronic high load (CHL)

**Fig. 3:**
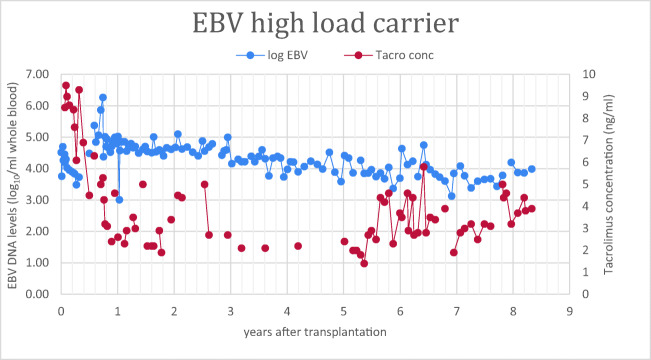
Prolonged EBV DNA CHL carrier state in one patient despite low level of immunosuppression. The characteristics of the patient (CHL6) are described in Table 4. He received a kidney from a deceased donor. Both donor and recipient were EBV seropositive before transplantation (D+R+). Immunosuppression was reduced to minimal dose of tacrolimus and steroids every other day. Mycophenolate mofetil was withdrawn 258 days after transplantation

Altogether, 14 children developed CHL, starting at a median of 69 days (0–278) post-transplant (Table 4). Eleven (79%) of the patients were EBV seronegative prior to transplantation and developed a primary EBV infection 40 days (26–82) post-transplant. Six patients developed CHL at the time when the primary EBV infection started, whereas five of the 11 patients developed CHL several months after the first positive EBV DNA. The remaining three patients developed CHL after a reactivation of EBV. All patients in the CHL group had their immunosuppression reduced because of high EBV loads. Target levels for tacrolimus were lowered to < 5 ng/ml followed by reduction of MMF, and because of persistent high EBV levels, MMF was withdrawn in five patients in this group. The maximum peak of EB viral load was 5.4 (4.7–6.1) log_10_ Geq/ml for the whole CHL group. The duration of CHL carriage varied between 0.5 and 6.5 years (median 2.0). Twelve children were CHL carriers for more than 1 year despite minimal immunosuppression. Three patients in the CHL group were treated for mild to moderate (one mild and two moderate) rejections with resolution of rejection. CHL-carriage was ongoing in two patients at the end of the study who had then experienced the CHL state for 7 and 78 months respectively. An example of prolonged high EBV DNA load despite low tacrolimus concentration (case CHL 6 in Table 4) is shown in Fig. 3.

**Table 4:** Characteristics of chronic high load carriers (CHL)

*Patient*	*Age at tx (years)*	*Gender*	*EBV serology at tx*	*Post-tx time to EBV start (days)*	*Post-tx, time to CHL start (days)*	*CHL Duration (months)*	*Maximum EBV load (log*_*10*_ *Geq /ml)*	*EBV-related symptoms at infection*	*Post-tx acute cellular rejection (days)*	*Post-tx follow-up time (years)*
*CHL1*	1.7	M	D+R−	47	54	> 7 *(ongoing)*	6.1	Yes, fever, pharyngitis, diarrhea	No	0.7
*CHL2*	1.5	M	D+R+	47	78	21	5.8	Yes, fever, pharyngitis, enlarged lymph nodes, PTLD investigated	No	3
*CHL3*	7	F	D+R−	40	210	29	4.9	Yes, neutropenia, PTLD investigated	No	5
*CHL4*	8	M	D+R−	82	231	17	5.2	No	No	6
*CHL5*	1.3	M	D+R−	68	68	> 78 *(ongoing)*	5.8	Yes, sub febrile	No	7
*CHL6*	2	M	D+R+	0	0	77	6.1	Yes, neutropenia, diarrhea, PTLD investigated	Yes,102 mild	8
*CHL7*	1.3	M	D+R−	42	60	15	5.2	Yes, diarrhea, vomiting	No	8
*CHL8*	2	M	D+R−	40	68	56	5.8	No	No	8
*CHL9*	9	F	D+R−	26	278	15	4.7	No	No	9
*CHL10*	1.1	M	D+R−	26	54	55	6.1	Yes, diarrhea, vomiting, pharyngitis	No	10
*CHL11*	15	F	D?R−	42	70	17	4.8	No	No	3
*CHL12*	1.1	M	D+R+	51	67	55	5.6	No	No	11
*CHL13*	2	F	D+R−	33	243	6.4	4.9	Yes, fever	Yes, 38 moderate	13
*CHL14*	2	M	D+R−	33	275	36	5.25	No	Yes, > 1330, moderate	7

*M* male, *F* female, *D* donor serostatus, *R* recipient serostatus, *EBV start* first positive EBV DNA post-transplant

Children with CHL had a follow-up time of 7.4 years (0.6–12) after high EBV load was first found. They were younger at transplantation (HR 0.74 [95% CI 0.63 to 0.87], *p* = 0.0002) and had a higher rate of congenital anomalies of the kidney and urinary tract (CAKUT) as underlying renal diagnosis (HR 3.92 [95% CI 1.23 to 12.51], *p* = 0.021) compared to the children who did not develop CHL (Table 5). When adjusting for age, the difference between CHL and non-CHL group was not significant regarding CAKUT (*p* = 0.16).

**Table 5:** Univariate analysis of risk for CHL in cohort of our renal transplant recipients

Variable	Category	*n* (%)	HR (95% CI)	*P* value
Age at transplantation (years)	≤ 10	13 (44.8)		
	> 10	1 (3.4)	0.74 (0.63:0.87)	*0.0002*
Sex	Male	10 (34.5)		
	Female	4 (13.8)	0.34 (0.11:1.10)	0.072
CAKUT	No	4 (12.1)		
	Yes	10 (40.0)	3.92 (1.23:12.51)	*0.021*
HLA mismatch	0–2	5 (19.2)		
	3–4 (vs 0–2)	8 (34.8)	1.89 (0.62:5.79)	ns
	5–6 (vs 0–2)	1 (11.1)	0.56 (0.07:4.82)	ns
Living donor	No	1 (7.1)		
	Yes	13 (29.5)	4.61 (0.60:35.30)	ns
dialysis before tx	No	5 (21.7)		
	Yes	9 (25.7)	1.28 (0.43:3.81)	ns
Diagnosis	CAKUT	10 (40.0)		
	Hereditary disorders vs CAKUT	3 (16.7)	0.35 (0.10:1.28)	ns
	Acquired diseases vs CAKUT	1 (7.7)	0.16 (0.02:1.27)	0.084
EBV mismatch (D+R−)	No	3 (15.0)		
	Yes	10 (35.7)	2.56 (0.70:9.32)	ns
EBV serology in recipients	Positive	3 (11.1)		
	Negative	11 (35.5)	3.55 (0.99:12.76)	0.052

*CAKUT* congenital anomalies of the kidney and urinary tract, *CHL* chronic high load, *HLA* human leucocyte antigen, *tx* transplantation

The clinical presentation of EBV infection among the CHL carriers was in most cases unspecific or asymptomatic (Table 4). Eight patients presented symptoms or clinical findings that could be caused by EBV, while EBV DNAemia was detected without any associated symptoms of active infection in six individuals.

Twelve of the 14 patients in the CHL group received CMV-prophylaxis, including four CMV D−/R− who were considered to have an increased risk of contracting primary CMV-infection (such as having siblings in pre-school).

The median GFR 3 months post-transplant was 82 (51–114) in the CHL group and 67 ml/min/1.73 m^2^ (25–103) in the non-CHL group (*p* = 0.0016). One year after transplantation, GFR did not differ between the groups (Table 1).

Three patients in the CHL group (CHL 2, 3, and 6 in Table 4) with rapidly rising EBV DNA levels or high levels for a long time were carefully examined for signs of PTLD, as described in the “Methods.” No patient developed PTLD, and no patient was treated with rituximab during a median clinical follow-up of 7.8 years (0.7–13).

### CMV co-infections

Among our 58 patients, 35 (60%) were CMV seronegative prior to transplantation (Table 3). Of the 14 recipients who became EBV CHL carriers, five (36%) had primary CMV infection and three (21%) had reactivated CMV DNAemia, as compared to 8 (18%) and 9 (20%) children respectively, in the non-CHL group (Table 3).

Primary CMV before or at the same time as EBV DNAemia (+/−1 month) was seen in three patients (2 CHL and 1 non-CHL) in the cohort. One of these patients had a primary CMV infection at the same time as the EBV high load period started (Table 3).

Symptoms that could be caused by CMV infection/disease developed in six CHL and four non-CHL patients. Of these, only three patients received antiviral treatment against CMV infection, two had primary infections and one reactivated, all belonging to the non-CHL group. One of these patients had leucopenia, diarrhea, and proctitis, and therefore, CMV tissue invasive disease was highly suspected. In addition, three patients had both EBV- and CMV-related symptoms. These patients presented only mild forms of infection, except for the third patient who died 9 months after transplantation due to bacterial pneumonia and multi-organ failure while having high EBV DNA levels in blood and concomitant CMV DNAemia.

## Discussion

Our main findings were that as many as 24% of the children with kidney transplants developed a EBV chronic high load carrier state (CHL), that many were CHL carriers for long periods of time despite reduced immunosuppression, and that no patient developed PTLD during follow up. Based on these findings, we suggest that an early detection of EBV DNAemia due to frequent EBV load measurements can improve the possibilities to adapt immunosuppressive treatment and therefore possibly reduce complications in a long-term follow-up of pediatric renal transplant patients.

In the present study, the frequency of CHL carriers was higher than the 8% recently described by Yamada et al. [27], which to some extent might be due to the relatively small study group, but also to our more frequent EBV DNA measurements undertaken during the first years post-transplant. The median time to onset of CHL in our cohort was 69 days post-transplant which is shorter than the 104 days in a previous study [32]. The median CHL duration of 2 years is similar to the results of other studies, but the follow-up time of almost 8 years is longer than other comparable studies [18, 27, 33]. In a previous multicenter study, 2% of PTLD was reported following renal transplantation [34]. In our study of 58 children, there were no cases of PTLD, which also might be due to the limited study size. Our strength is that this is a single-center study where all patients were followed according to the same protocol, with frequent clinical check-ups by the same team of doctors and nurses and with close contact with the local hospital. In addition, all laboratory samples were analyzed with the same methods and with the same EBV DNA cut-offs.

Young age is a well-known risk factor for developing chronic high EBV load and PTLD [14, 34]. In our study, CHL was more frequent in young individuals and the children in the CHL-group were younger (median age 2 years) than described by Yamada et al. (median age 3.8 years) [27]. However, young age often coincides with being EBV seronegative which also predisposes for primary EBV infection and thereby also for CHL carriership and eventually PTLD. In our study, 53% of the patients were EBV seronegative before transplantation, which is similar to previous reports [27, 35, 36]. Eighty percent of our seronegative patients developed a primary EBV infection during the study period, which is higher than previous observations [18, 27]. As many as 74% of our EBV seropositive patients reactivated their EBV infection, which is a higher rate than observed by Colombini et al. [18] and Höcker et al. [37], and our higher EBV rates are likely explained by our lower EBV threshold for classification (200 copies/ml as compared with 3000 copies/ml and 1000 copies/ml, respectively, in their studies).

Green et al. [17, 38] have described the chronic high EB viral load as occurring more often after primary EBV infection and seldom as reactivated infections. Accordingly, in our study, out of 28 EBV mismatch (D+/R−) patients, 25 (89%) developed a primary EBV infection within 2 months after transplantation and 11 (44%) progressed to CHL. In our CHL group, 11 patients were seronegative at transplantation and had a primary EBV infection post-transplant. The remaining three children had their first positive test for EBV at or shortly before transplantation. They were classified as EBV reactivation according to protocol. Since they had an active primary infection with early EBV IgG production at the time of transplantation and the introduction of immunosuppression, they may have performed more similarly to the children with primary infection post-transplant. This may explain the lack of difference in time interval to first occurrence of EBV DNAemia, maximum EBV level, or duration of CHL between the patients with primary and those with reactivated EBV infections. Thus, most children who developed CHL experienced a primary EBV-infection post transplantation, but this was not a statistically significant independent risk factor in our study when adjusting for age.

The increasing number of young renal transplant recipients may account for a higher rate of EBV-seronegative individuals receiving transplants, and thus also for primary EBV infection after transplantation. The risk of primary EBV infection after pediatric renal transplantation might lead to an increased prevalence of EBV-associated PTLD that could become a rising problem in the future [39].

In our group of 14 patients with CHL, we noted a higher rate of CAKUT (*p* = 0.021) as pre-transplant renal diagnosis, which can be explained by young age at transplantation in patients with these diagnoses. When adjusting for age, there was no significant difference of CAKUT between the CHL and non-CHL groups. CAKUT are more common in boys and, although not significant, there was a trend of more boys in the CHL group.

Immunosuppression is another risk factor for developing CHL- and EBV-associated PTLD [16, 22, 25, 37]. Thus, the incidence of PTLD is higher in pediatric recipients of heart, lung, and intestinal transplants, traditionally treated with more intense immunosuppression, and lower in pediatric liver and renal recipients, who are treated with less intense immunosuppression [6, 17, 40]. In the present study, all children followed the same initial immunosuppressive protocol. Twenty-seven children (14 CHL and 13 non-CHL) had their immunosuppression reduced or MMF withdrawn because of increasing or maintaining high EBV DNA levels. Since the CHL children in our study were younger than the non-CHL children, their relatively immature immune systems without fully developed T-cell-response could have played a role. Thus, younger children might be over-immunosuppressed, which could have contributed to the development of CHL. Immunosuppression was increased in some specific situations, such as multiple HLA-mismatches between donor and recipient or after acute cellular rejection, implying an increased risk for CHL and PTLD. On the other hand, there was a fear that the reduced immunosuppression in the CHL group would increase the incidence of rejections. Since no such increase of rejections was observed, the EBV CHL carriage and PTLD may be a manifestation of over-immunosuppression [16, 17, 25, 37].

There is no universally accepted approach for the management of post-transplant EBV infections. Reduction of the total burden of immunosuppression is one therapeutic option. In the present study, immunosuppression was reduced in all 14 patients in the CHL group and in several of the non-CHL patients due to short-term high EBV loads. The use of antiviral agents to prevent EBV infection in pediatric patients with EBV seroconversion is another, but controversial, option [6, 38, 41]. No additional antiviral therapy was applied in our patients.

Eight of the 14 CHL patients were also co-infected with CMV. A similar frequency was seen in the non-CHL group. Primary CMV infections were seen in 36% in the CHL-group compared to 18% in the non-CHL group. This is consistent with the results of previous investigations where primary CMV infection is described as a risk factor for CHL and PTLD [7, 23, 42]. In our study, we observed that CMV DNAemia arose in 64% of the children after antiviral prophylaxis was ceased. Antiviral prophylaxis has been shown to reduce the burden of CMV while postponing its occurrence until after prophylaxis cessation [43, 44]. Hence, our data supports the importance of assessing CMV DNA also after antiviral prophylaxis has been ceased.

EBV viremia may be asymptomatic or related to non-specific symptoms of infection. Of the 58 studied patients, 18 (31%) developed symptoms during follow up that could have been caused by EBV or CMV infection. The clinical presentation of the patients with EBV infection ranged from vague gastrointestinal complaints to fulminant multi-organ failure. Eight (57%) of the CHL patients had symptoms or clinical findings that could be attributed to EBV, as described in the “Results” and specified in Table 4. As in previous studies, the difficulty of attributing symptoms to EBV, CMV, or other viral and bacterial infections is a limitation. For example, it could not be established to what extent EBV or CMV contributed to death 9 months post-transplant in one of our patients.

High EBV DNA levels have been recognized as a risk factor for developing PTLD in transplant recipients [2, 22, 45]. However, children may remain in a CHL carrier state without developing PTLD, and high or persistent EBV load alone does not appear to be predictive for development of PTLD in pediatric renal transplant recipients [25, 46]. On the other hand, EBV-associated PTLD has been reported to occur in 1–7% of pediatric renal transplant recipients [13, 15]. Despite the association between EBV infection and PTLD, there is still no consensus on viral load monitoring or the benefits of EBV antiviral prophylaxis/treatment. Better understanding of the relationship between EBV viral load and the risk of developing PTLD after renal transplantation is required [2, 25]. Risk factors of potential importance are the intensity of immunosuppressive therapy [30, 37], the EBV virulence [47], the nature of EBV-infected B-cells [48], the EBV-specific T-cell response [49], and a genetic predisposition [50].

A previous study did observe an association between sub-clinical EBV infection and impaired graft function in pediatric renal transplant patients [51]. In our study, as well as in another previous study by Höcker et al. [37], no significant difference in GFR decline was found between patients with or without EBV infection. In our study, the GFR 3 months after transplantation was higher in the CHL-group compared to the non-CHL-group, probably because of the patients’ lower age. However, to detect any significant impact of EBV-infection on graft survival, a larger prospective study is needed.

The present study suggests that close monitoring of EBV DNA levels post-transplant can facilitate the identification of patients at risk of developing CHL and PTLD. Because early primary EBV infection is a risk for chronic EBV-associated diseases, avoiding primary infection post-transplant would be important, but is difficult since most organ donors are EBV seropositive, and because exposure to EBV is frequent at an early age.

## Conclusion

CHL carriage after renal transplantation occurred frequently (24%), was often long lasting, and developed mainly in younger children. The absence of PTLD in our patients suggests that monitoring of EBV DNA to guide immunosuppression may be effective in reducing the risk of PTLD. Future studies should aim at identifying additional risk factors for PTLD to modify the intensity and duration of monitoring. The increasing number of renal transplantations at younger age will probably lead to higher rates of primary EBV-infection and CHL in recipients in the future. Understanding the risk of EBV load in different organ transplant settings will aid clinical decisions regarding immunosuppression levels when balancing the risk of rejection and infection.

## Abbreviations

CAKUT: Congenital anomalies of the kidney and urinary tract
CHL: Chronic high viral load
CMV: Cytomegalovirus
CV: Coefficient of variation
D/R: Donor/recipient
EBV: Epstein-Barr virus
ELISA: Enzyme-linked immunosorbent assay for detection of antibodies
Geq: Genome equivalents
GFR: Glomerular filtration rate
HLA: Human leucocyte antigen
LVL: Low viral load
MMF: Mycophenolate mofetil
PCR: Polymerase chain reaction
PTLD: Post-transplant lymphoproliferative disorder
SOT: Solid organ transplantation
Tx: Transplantation, transplant
UVL: Undetectable viral load
